# Head-to-head comparison of sirolimus- versus paclitaxel-coated balloon angioplasty in the femoropopliteal artery: study protocol for the randomized controlled SIRONA trial

**DOI:** 10.1186/s13063-021-05631-9

**Published:** 2021-09-28

**Authors:** Ulf Teichgräber, Maja Ingwersen, Stephanie Platzer, Thomas Lehmann, Thomas Zeller, René Aschenbach, Dierk Scheinert

**Affiliations:** 1 Department of Radiology, Jena University Hospital, Friedrich-Schiller-University Jena, Jena, Germany; 2 Center for Clinical Studies, Jena University Hospital, Friedrich-Schiller-University Jena, Jena, Germany; 3 Department of Angiology, Universitäts-Herzzentrum Freiburg-Bad Krozingen, Bad Krozingen, Germany; 4 Department of Angiology, University Hospital Leipzig, Leipzig, Germany

**Keywords:** Balloon angioplasty, Femoral artery, Paclitaxel, Popliteal artery, Peripheral artery disease, Quality of life, Sirolimus, Randomized controlled trial

## Abstract

**Background:**

Endovascular revascularization has established as the first-line therapy of femoropopliteal artery disease. Paclitaxel-coated balloon angioplasty proved to be superior to plain old balloon angioplasty (POBA) regarding prevention of restenosis and need for recurrent revascularization. Over the past years, paclitaxel was the only active drug to inhibit neointimal proliferation which could be processed to an appropriate balloon coating. The purpose of this study is to assess whether efficacy and safety of sirolimus-coated balloon angioplasty is noninferior to paclitaxel-coated balloon angioplasty.

**Methods:**

This randomized controlled, single-blinded, multicentre, investigator-initiated noninferiority trial aims to enrol a total of 478 participants with symptomatic femoropopliteal artery disease of Rutherford category 2 to 4 due to de novo stenosis or restenosis. After pre-dilation, participants will be allocated in a 1:1 ratio to either sirolimus- or paclitaxel-coated balloon angioplasty. Post-dilation with the drug-coated balloon (DCB) used or standard balloon is mandatory in case ≥ 50%, and optional in case of ≥ 30% residual diameter stenosis. Bailout stenting with bare-metal nitinol stents should be conducted in case of flow-limiting dissection. Primary noninferiority endpoints are primary patency and the composite of all-cause mortality, major target limb amputation, and clinically driven target lesion revascularization at 12 months. Secondary outcomes are clinical and hemodynamic improvement, change in health-related quality of life, and safety throughout 60 months.

**Discussion:**

Although concerns about long-term safety of paclitaxel-coated devices were not confirmed by recent patient-level data analyses, conflicting evidence contributed to a loss of confidence among patients and physicians. Therefore, sirolimus, known for a broader therapeutic range than paclitaxel, may serve as a welcome alternative. This will be justified if noninferiority of sirolimus-coated balloon angioplasty against the current standard of paclitaxel-coated balloon angioplasty can be demonstrated.

**Trial registration:**

ClinicalTrials.govNCT04475783. Registered on 17 July 2020

EUDAMED No. CIV-20-11-035172, DRKS00022452

## Administrative information

Note: the numbers in curly brackets in this protocol refer to SPIRIT checklist item numbers. The order of the items has been modified to group similar items (see http://www.equator-network.org/reporting-guidelines/spirit-2013-statement-defining-standard-protocol-items-for-clinical-trials/).

**Table:** 

Title {1}	Head-to-head comparison of sirolimus- versus paclitaxel-eluting balloon angioplasty in the femoropopliteal artery
Trial registration {2a and 2b}.	www.ClinicalTrials.gov NCT04475783 EUDAMED No: CIV-20-11-035172 DRKS00022452
Protocol version {3}	18 November 2020; V02.1
Funding {4}	The study receives funding by the manufacturer of the investigational product, Concept Medical Inc., Tampa, Florida, USA. The manufacturer also provides the investigational product (Magic Touch® PTA sirolimus drug-coated balloon) for index procedures free of charge.
Author details {5a}	Prof. Dr. Ulf Teichgräber, Department of Radiology, Jena University Hospital, Friedrich-Schiller-University Jena, Germany Dr. Maja Ingwersen, Department of Radiology, Jena University Hospital, Friedrich-Schiller-University Jena, Germany Dr. Stephanie Platzer, Center for Clinical Studies, Jena University Hospital, Friedrich-Schiller-University Jena, Germany Dr. Thomas Lehmann, Center for Clinical Studies, Jena University Hospital, Friedrich-Schiller-University Jena, Germany Prof. Thomas Zeller, Department of Angiology, Universitäts-Herzzentrum Freiburg-Bad Krozingen, Bad Krozingen, Germany PD Dr. René Aschenbach, Jena University Hospital, Friedrich-Schiller-University Jena, Germany Prof. Dierk Scheinert, Department of Angiology, University Hospital Leipzig, Leipzig, Germany
Name and contact information for the trial sponsor {5b}	Friedrich-Schiller-University, Jena, Germany Sponsor Representative: Prof. Dr. Ulf Teichgräber, Jena University Hospital, Department of Radiology, Am Klinikum1, 07747 Jena, Phone: +49 3641 9324831, Fax: +49 3641 9324832 ulf.teichgraeber@med.uni-jena.de
Role of sponsor {5c}	The study sponsor (Friedrich-Schiller-University Jena) will have ultimate authority over study design; collection, management, analysis, and interpretation of data; writing of the report; and the decision to submit the report for publication.

## Introduction

### Background and rationale {6a}

Lower limb peripheral arterial disease (PAD) is a common syndrome that affects an estimated 27 million adults in Europe and North America. PAD is associated with significant morbidity and mortality. Prevalence ranges from 3 to 10%, however, increases with age up to 20% in individuals older than 70 [1].

Symptomatic PAD initially presents as claudication and may progress into chronic limb-threatening ischemia (CLTI), defined as presence of rest pain in the affected limb and/or tissue loss (ulcers, gangrene). Mortality in CLTI patients is 20% in the first year after presentation. Long-term data suggest an increase of mortality up to 50% at 5 years [1].

Percutaneous transluminal balloon angioplasty (PTA) has become the standard treatment for PAD. However, long-term results after plain old balloon angioplasty (POBA) are hampered by the occurrence of re-obstruction of the treated segment due to intimal hyperplasia [2] and stents are associated with a number of potential disadvantages such as occurrence of in-stent restenosis, hindrance of later surgical revascularization, and need for prolonged antiplatelet therapy [3]. Concerns exist about stent fractures and their clinical implications [4]. Based on these limitations, drug-coated balloons (DCB) came into focus.

Most of the investigated DCBs are coated with paclitaxel which disrupts normal microtubule function and prevents neointimal hyperplasia by inhibiting smooth muscle cell migration, proliferation, and extracellular matrix secretion [5]. Several paclitaxel-coated balloon types with various excipients and different dose densities of paclitaxel demonstrated superiority to POBA [6–10]. While these prior studies clearly showed efficacy of DCB regarding prevention of restenosis and target lesion revascularization (TLR), a recent meta-analysis showed an increased 2-year mortality after peripheral DCB angioplasty [11]. However, underlying assumptions for a dose-response relationship gave rise to controversy [5] and subsequent research based on patient-level data refuted the paclitaxel dose argument [12, 13]. So far, regulatory agencies including the German Federal Institute for Drugs and Medical Devices (BfArM) recommend a careful discussion of risks and benefits of DCB treatment with all patients and demand safety monitoring of patients who have been treated with paclitaxel-coated balloons. Considering potential risks associated with paclitaxel-coated balloons, it seems reasonable to look for alternative treatment approaches.

We initiated the randomized controlled SIRONA study to compare efficacy and safety of a commercially marketed sirolimus drug-coated balloon with established paclitaxel drug-coated balloons. The rationale of this study is based on the hypothesis that angioplasty by means of the Magic Touch® PTA sirolimus-coated balloon catheter (Concept Medical Inc., Tampa, USA) is noninferior to paclitaxel-coated balloon angioplasty, which would imply that it could be considered an alternative approach to paclitaxel-coated balloon angioplasty.

## Objectives {7}

### Primary objectives

1. Primary efficacy objective of our study is to assess whether primary patency at 1-year after Magic Touch® Sirolimus-coated balloon angioplasty of femoropopliteal lesions is noninferior to that after paclitaxel-coated balloon angioplasty. Noninferiority margin is set to 10%. Primary patency is defined as freedom from restenosis (> 50% diameter stenosis evidenced by peak systolic velocity ration [PSVR] > 2.4 by duplex ultrasound [DUS] without the need for target lesion revascularization).
2. Primary safety objective is to assess whether freedom from the composite of clinically driven target lesion revascularization (TLR), major target limb amputation, and all-cause mortality at 1 year after Magic Touch® Sirolimus-coated balloon angioplasty of femoropopliteal lesions is noninferior to that after paclitaxel-coated balloon angioplasty. Noninferiority margin is set to 10%.

### Secondary objectives

Secondary objectives are to compare hemodynamic improvement, morphological outcome, clinical improvement, change in health-related quality of life, and safety throughout 5 years after paclitaxel- or sirolimus-coated balloon angioplasty.

## Trial design {8}

**Fig. 1:**
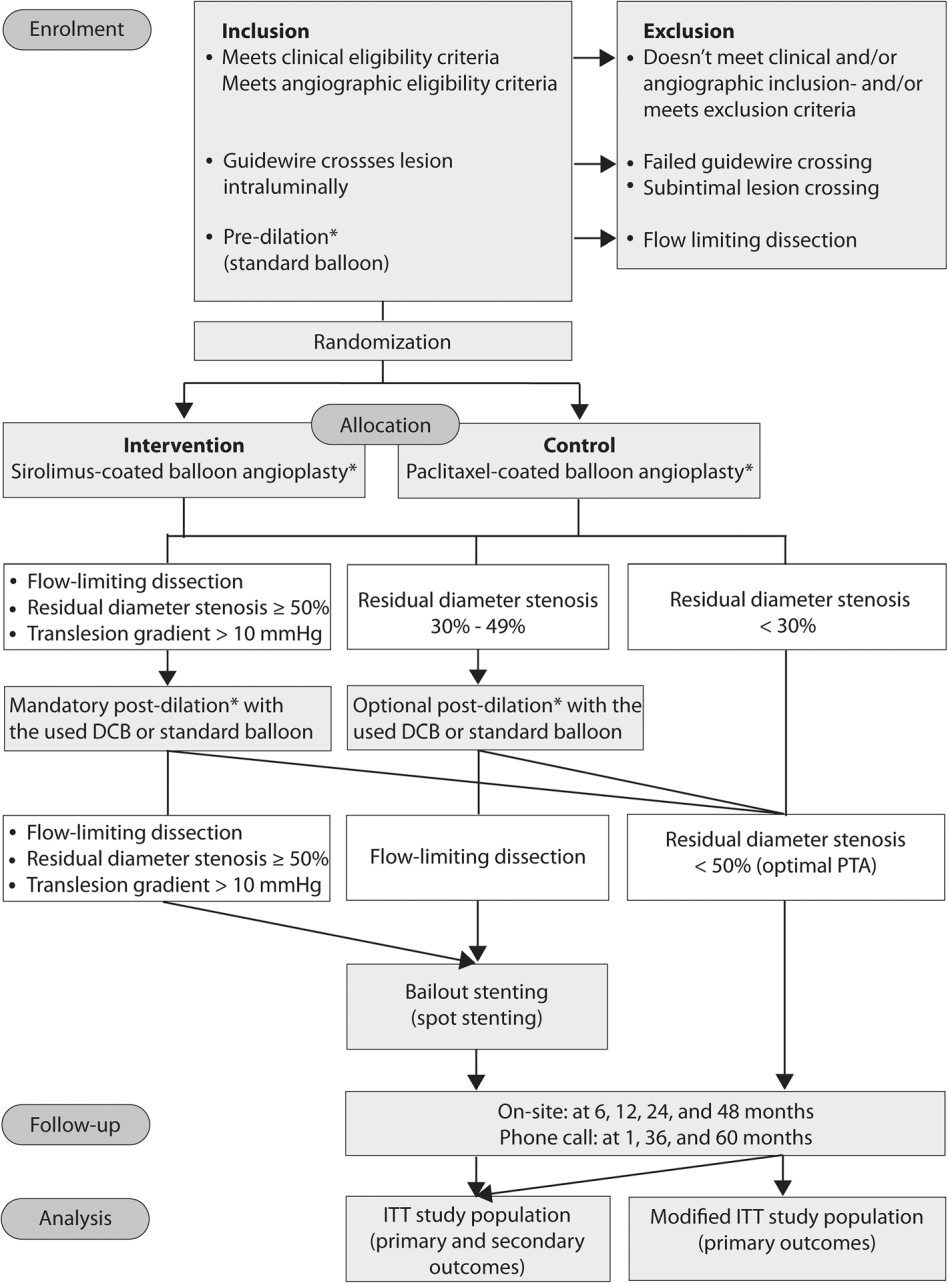
Study flow chart. Asterisk indicates pre-dilation, drug-coated balloon dilation, and post-dilation should last for at least 60 s each. Dilation for 180 s each is strongly recommended. DCB, drug-coated balloon; PTA, percutaneous transluminal angioplasty; ITT, intention to treat

The SIRONA study (head-to-head comparison of SIROlimus versus paclitaxel drug-eluting ballooN Angioplasty in the femoropopliteal artery) is designed as randomized controlled, single-blinded, parallel group, multicentre, investigator-initiated, noninferiority, trial. Allocation ratio of participants is set at 1:1 (Fig. 1). The Friedrich-Schiller-University ethics committee, Jena, Germany, approved the study (Reg.-Nr. 2020-2012-MPG_ff, dated 11 February 2021). In addition, written permissions from respective local ethics committees have to be obtained by all participating sites. All study devices have a European certificate of conformity (CE mark). The study is registered with ClinicalTrials.gov (NCT04475783) and with the German clinical trials register (DRKS00022452).

## Methods: Participants, interventions and outcomes

### Study setting {9}

About 25 study sites including university hospitals, community, and outpatient clinics in Germany and Austria will participate in the SIRONA study. The list of study centres can be obtained from continuously updated entries on ClinicalTrials.gov.

### Eligibility criteria {10}

#### Inclusion criteria

Individuals are eligible for trial participation if the following criteria apply:

1. Age ≥ 18 years
2. Clinical symptoms meet Rutherford category 2 to 4
3. Single de novo or re-stenosed lesion of the superficial femoral artery (SFA) and/or the proximal segment (P1) of the popliteal artery
4. Target lesion diameter stenosis of ≥ 70% assessed by angiography
5. Target lesion length of ≥ 2 cm and ≤ 20 cm by visual estimate
6. Multiple lesions with a healthy vessel segment of maximum 3 cm in between can be considered single lesion at discretion of the operator (total lesion length should not exceed 20 cm)

##### Reference vessel diameter (RVD) ≥ 4 mm and ≤ 6.5 mm by visual estimate

7. Patency of ipsilateral iliac artery (≤ 30% diameter stenosis). Iliac artery stenosis > 30% may be treated during the index procedure to ensure sufficient inflow
8. Patency of P2 and P3 segment of the popliteal artery and at least one (1) infrapopliteal artery (< 50% diameter stenosis) ensuring sufficient outflow from the femoropopliteal artery
9. Guidewire has successfully crossed the target lesion intraluminally
10. Pre-dilation of the target lesion
11. Participants can only be enroled once with a single target lesion
12. Participant’s declaration of informed consent

#### Exclusion criteria

Individuals are excluded from trial participation if any of the following criteria apply:

1. Subintimal or failed guidewire crossing of the target lesion
2. Flow-limiting dissection after pre-dilation of the target lesion
3. Angiographic evidence of severe calcification of the target vessel (contiguous calcification on both sides of the vessel)
4. Presence of fresh thrombus in the target lesion
5. Presence of aneurysm in the target vessel
6. In-stent restenosis of the target lesion
7. Prior vascular surgery of the target limb
8. History of major amputation in the target limb
9. Any vascular surgical procedure or intervention performed in the target limb within 30 days prior to or planned within 30 days post index procedure
10. Any vascular treatment with paclitaxel- or sirolimus-coated devices within 60 days prior to index procedure
11. Vascular disease in the opposite leg that requires treatment at the time of index procedure
12. Target lesion requires treatment with alternative therapies such as primary stenting, laser, lithotripsy, thrombectomy, atherectomy, cryoplasty, brachytherapy, and re-entry devices
13. Stroke or heart attack within 3 months prior to enrolment
14. Known allergies or sensitivity to heparin, aspirin, other anticoagulant/antiplatelet therapies, sirolimus, paclitaxel, or contrast media that cannot be adequately pre-treated prior to index procedure
15. Significant gastrointestinal bleeding or any coagulopathy that would contraindicate antiplatelet therapy
16. Dialysis or immunosuppressant therapy
17. Pregnant or lactating women
18. Life expectancy of less than 1 year in the opinion of the investigator
19. Participant enroled in another investigational drug, device, or biologic study

#### Eligibility criteria for participating sites and investigators

Participating sites must be equipped with the appropriate resources to meet the study requirements and shall have access to emergency units to perform bypass surgery in case of failed PTA. Investigators who will conduct the interventions are eligible if they are radiologists or angiologists with sufficient experience in the field of peripheral endovascular interventions.

#### Who will take informed consent? {26a}

Prior to inclusion and in accordance with the Declaration of Helsinki and national regulations, patients must undergo the consent process. During the consent process, the investigator or his/her designee must fully inform the patient about all relevant study details including potential risks and benefits of participation. Written informed consent is prerequisite for inclusion into the study.

### Additional consent provisions for collection and use of participant data and biological specimens {26b}

Not applicable.

## Interventions

### Explanation for the choice of comparators {6b}

Patients allocated to the control group will be treated with a paclitaxel-coated balloon catheter. Only those commercially available paclitaxel-coated balloon types are permitted for use in the study of which 2-year results from randomized controlled trials on obstructive PAD have been published.

The following DCB types are available for selection upon investigator’s discretion and to be used according to manufacturer’s instruction for use:

- IN.PACT® Admiral® (Medtronic, Minneapolis, USA)
- Luminor 35® (iVascular, Barcelona, Spain)
- Lutonix® (BD BARD Peripheral Vascular, Inc., Tempe, USA)
- Orchid® (Acotec Scientific Co., Ltd. Beijing, China)
- Ranger® (Boston Scientific, Voisins-le-Bretonneux, France)
- SeQuent® Please OTW (B. Braun Melsungen AG, Melsungen, Germany)
- Stellarex® (Philips, Amsterdam, The Netherlands)

Even in the control group, pre-dilation is mandatory and has to be performed with a conventional, undersized, non-drug-coated angioplasty balloon. Nominal balloon diameter has to be 1 mm smaller than the distal reference vessel diameter (RVD). Pre-dilation should last at least 60 s; however, prolonged pre-dilation for 180 s is strongly recommended.

### Intervention description {11a}

After angiographic assessment of the lesion and successful intraluminal guide wire crossing, pre-dilation of the target lesion with non-drug-coated conventional balloon for at least 60 s is mandatory. Prolonged pre-dilation for 180 s is strongly recommended. Pre-dilation balloon should be 1 mm smaller in diameter than the distal RVD. In case of flow-limiting dissection due to pre-dilation, participants will be excluded. Allocation to study treatment will be done immediately after pre-dilation.

Nominal diameter of DCB should match the distal RVD and length has to exceed each end of the target lesion by about 1 cm. Balloon inflation pressure should be at least nominal pressure but must not exceed rated burst pressure. Inflation should be maintained for a minimum of 60 s. Prolonged inflation for 180 s is strongly recommended. If more than one DCB is necessary for complete lesion coverage, multiple DCBs should overlap by at least 1 cm.

Post-dilation of at least 60 s with the DCB used or a standard uncoated balloon should be conducted in case of residual diameter stenosis of ≥ 50% by visual estimate, or > 10 mmHg trans-lesion gradient, or flow-limiting dissection. Prolonged dilation for 180 s is strongly recommended. In case of 30 to 49% residual diameter stenosis, repeated dilation is at investigator’s discretion. It is recommended to perform focal post-dilation with standard balloons of minimal length, sufficient to just cover the residual stenotic segment strictly within the treated area. Prolonged or repeated post-dilation should aim at < 50% diameter residual diameter stenosis by visual estimate without stenting (optimal PTA). DUS of the target lesion and determination of ankle brachial index (ABI) have to be performed before discharge and within 2 working days after index procedure.

### Criteria for discontinuing or modifying allocated interventions {11b}

In case of PTA failure after DCB angioplasty and post-dilation, defined as residual diameter stenosis of ≥ 50% by visual estimate, or > 10 mmHg trans-lesion gradient, or flow-limiting dissection despite the attempt of optimal PTA, bailout stenting should be performed. Bailout stenting should be conducted as spot stenting, which means utilization of as few and as short as possible stents to cover the residual stenosis. Only self-expanding, uncovered, bare nitinol stents are permitted for bailout stenting. Participants who underwent bailout stenting or emergency bypass surgery will be followed up per protocol and included into the intention to treat (ITT) analysis. If emergency bypass or other target limb surgical intervention is required during index procedure, participants will be excluded from study participation.

### Strategies to improve adherence to interventions {11c}

Not applicable.

#### Relevant concomitant care permitted or prohibited during the trial {11d}

Antiplatelet therapy has to be used in both study arms according to clinical routine. Prior to the index procedure, it is strongly recommended to administer dual antiplatelet therapy (DAPT) as a combination of aspirin (100 mg daily at least 3 days before procedure or a loading dose of 500 mg) and clopidogrel (75 mg daily at least 3 days before procedure or a loading dose of 300 mg), or aspirin and rivaroxaban (2.5 mg twice a day) per hospital standard of care. During index procedure, participants must receive appropriate anticoagulation by means of heparin according to the institution’s standard of care. DAPT is recommended for at least 4 weeks after index procedure (aspirin 100 mg daily, clopidogrel 75 mg daily) and single antiplatelet therapy indefinitely thereafter (aspirin 100 mg daily) according to centres’ standard of care.

No concomitant approach of revascularization using drug-eluting stents, covered stents, laser atherectomy, cryoplasty, re-entry devices, cutting or scoring balloons, brachytherapy, or non-study device DCBs is permitted.

### Provisions for post-trial care {30}

Not applicable.

### Outcomes {12}

The assessment schedule is presented in Table 1.

**Table 1:** Schedule of enrolment, interventions, and assessments

Study period												
	Index procedure											
	Enrolment		Allocation	Angioplasty	Post-procedure	Follow-up						
Timepoint	***-t***_***1***_^a^	***-t***_***2***_^b^	0	Immediately after allocation	Before discharge^§^	1-month ± 7 days Phone call	6-month ± 30 days On-site	12-month ± 45 days On-site	24-month ± 60 days On-site	36-month ± 60 days Phone call	48-month ± 60 days On-site	60-month ± 60 days Phone call
**Enrolment**												
**Eligibility screen**	X											
**Informed consent**	X											
**Allocation**			X									
**Interventions**												
**Intraluminal guide wire crossing**^c^		X										
**Pre-dilation**^e^		X										
**Sirolimus-coated balloon angioplasty**				X								
**Paclitaxel-coated balloon angioplasty (control)**				X								
**Assessments**												
**Demographic data**	X											
**Physical examination**	X				X		X	X	X		X	
**Laboratory examination**^f^	X											
**Medical history**	X											
**Concomitant medication**				X	X	X	X	X	X	X	X	X
**Angiography**		X		X								
**Duplex ultrasound**^g^					X		X	X	X		X	
**ABI**	X				X		X	X	X		X	
**Rutherford category**	X						X	X	X		X	
**Walking distance (participant self-assessment**^h^**)**	X					X	X	X	X	X	X	X
**VascuQuol Score**	X						X	X	X		X	
**6-min walk test**	X						X	X	X		X	
**Treadmill test (optional)**	X						X	X	X		X	
**EQ5D-3L index**	X					X	X	X	X	X	X	X
**AE/SAE**				X	X	X	X	X	X	X	X	X

^a^At baseline

^b^During index procedure

^c^Successful intraluminal guidewire crossing of the lesion

^d^Within 2 working days after index procedure and before discharge

^e^Pre-dilation without flow-limiting dissection

^f^Including pregnancy test were appropriate

^g^DUS should also be performed before and after any target vessel revascularization. Adjudication by core laboratory at 6, 12, and 24 months

^h^Participant self-assessment of maximum walking distance should be performed before walk tests and questionnaires

*AE* adverse event, *SAE* severe adverse event

#### Primary outcomes

- Primary efficacy outcome is the incidence of primary patency at 1 year. Primary patency is defined as absence of target lesion restenosis assessed by duplex ultrasound (restenosis indicated by peak systolic velocity ratio [PSVR] > 2.4 and adjudicated by core laboratory) without the need for recurrent target lesion revascularization. If 1-year primary patency in the intervention group is no less than 10 percentage points worse than that in the control group, it will be considered noninferior. Clinical relevance of the primary outcome is that loss of primary patency often leads to symptoms and necessitates TLR. In addition, primary patency can be objectively assessed by investigators with DUS and independently confirmed by the blinded core laboratory.
- Primary safety outcome is the composite of freedom from all-cause mortality, major target limb amputation, and clinically driven TLR at 1 year. Sirolimus-coated balloon angioplasty will demonstrate noninferiority when 1-year incidence of the primary safety outcome is no worse than that of the control group by more than 10 percentage points.

Primary outcomes will be determined for both the ITT and the modified ITT (only participants who received the assigned treatment) study population (Fig. 1).

#### Secondary outcomes

- Hemodynamic improvement defined as increase in resting ankle brachial index (ABI) by ≥ 0.15 or to ≥ 0.9 from pre-procedure without the need for target vessel revascularization (TVR) or amputation
- Binary restenosis (PSVR > 2.4 assessed with DUS and adjudicated by core laboratory)
- Primary patency (Kaplan-Meier estimate) at 6, 24, and 48 months
- Secondary patency (Kaplan-Meier estimate)
- Clinical improvement by at least one Rutherford category without the need for target vessel revascularization (TVR) or amputation
- Kaplan-Meier estimate of clinically driven target lesion revascularization (TLR) due to symptoms or ABI drop of ≥ 20% or > 0.15 from post-procedure and restenosis determined by angiography and/or indicated by PSVR > 2.4 assessed with DUS and adjudicated by core laboratory (not including procedural bailout stenting)
- Maximum walking distance determined by participant self-assessment
- Vascular quality of life score (VascuQoL)
- Walking distance assessed by 6-min walk test
- Optional: maximum walking distance determined with treadmill test
- Health-related quality of life (European Quality of Life 5 Dimensions 3 Level [EQ-5D-3L] instrument)
- Secondary safety outcome is the composite of freedom from all-cause mortality, major target limb amputation, and clinically driven TLR at 5 years.

Secondary outcomes will be reported at 1, 6, 12, 24, 36, 48, and 60 months. As follow-up at 1, 36, and 60 months will only be conducted by phone, only participant self-assessed walking distance, EQ5D-3L index, and secondary composite safety outcome will be assessed at these times.

### Participant timeline {13}

Enrolment is expected to take 24 months and participants will be followed up through 60 months.

After index procedure, follow-up on-site visits will be performed at 6, 12, 24, and 48 months and follow-up phone calls at 1, 36, and 60 months. Participant timeline is specified in Table 1.

### Sample size {14}

In the randomized controlled pilot COMPARE trial, 1-year primary patency after paclitaxel-coated balloon angioplasty (IN.PACT DCB) was 89.0% [14]. In addition, the IN.PACT global study revealed 94% freedom from the primary composite safety endpoint in the long-lesion imaging cohort [15].

From this, we calculated that 430 participants (215 in each group) need to be analysed regarding the primary efficacy outcome, and 280 (140 in each group) regarding the primary safety outcome to show noninferiority of the Magic Touch sirolimus DCB over paclitaxel-coated balloon angioplasty with a power of 89% for every single test and of 80% for both primary outcomes. Noninferiority margin is set at 10% for both outcomes.

As heterogeneity regarding 12-month primary patency and need for TLR (the main driver of the primary composite safety endpoint in terms of quantity) is substantial even across different types of paclitaxel-coated balloons (65 to 86% [*I*^2^ = 54%] and 71 to 93% [*I*^2^ = 66%], respectively [16]); a deviation from the average by 10% with sirolimus-coated balloon angioplasty would still be within the range that can be considered noninferior.

One-sided Farrington-Manning test will be applied for the primary outcomes. Finally, assuming a dropout rate of 10%, 478 patients (239 per group) should be recruited.

### Recruitment {15}

Adequate participant enrolment within 24 months should be ensured by the high prevalence of peripheral artery disease of up to 20% depending on age, and the substantial number of about 25 participating sites.

### Assignment of interventions: allocation

#### Sequence generation {16a}

Enroled subjects will be randomized either to Magic Touch DCB angioplasty (intervention group) or to paclitaxel DCB angioplasty (control arm) at a ratio of 1:1. Randomization will be restricted by randomly varying block size and stratification by centre. A computer-generated randomization list will be prepared by an independent statistician not involved in enrolment and analyses using the software nQuery Advisor.

### Concealment mechanism {16b}

For concealment, sealed envelopes containing the treatment assignment will be provided by the Centre for Clinical Studies in Jena.

### Implementation {16c}

Allocation sequence will be generated by an independent statistician who is not involved in enrolment and analyses. Investigators will enrol participants upon ascertained eligibility and successful intraluminal lesion crossing of the guide wire. Subsequently, assignment to interventions will be conducted by investigators according to randomization.

## Assignment of interventions: blinding

### Who will be blinded {17a}

Participants but not investigators will be blinded to the study treatment. Additionally, core laboratory assessment of angiographic und DUS imaging will be blinded.

### Procedure for unblinding if needed {17b}

Not applicable.

## Data collection and management

### Plans for assessment and collection of outcomes {18a}

- *Angiography*. Before the index procedure and prior to any TVR, angiographic assessment must be performed. Angiographic imaging (X-ray and digital subtraction angiography) will be reviewed by a blinded core laboratory.
- *Duplex ultrasound*. PSVR > 2.4 indicates diameter restenosis of > 50% [17] and loss of primary patency. DUS will be adjudicated by blinded core laboratory assessment at 6, 12, and 24 months.
- *Rutherford category*. Clinical improvement is present in case of improvement by at least one Rutherford category according to current guidelines [18] without the need for TVR.
- *Ankle brachial index*. ABI will be determined according to current guidelines [18]. Hemodynamic improvement is present in case of ABI increase by at least 1.5 or to 0.9 ABI without the need for TVR.
- *Walking distance (participant self-assessment)*. Before other walking assessments, participants will be asked about their actual self-estimated maximum walking distance.
- *Vascular quality of life questionnaire (VascuQoL)*. VascuQoL as PAD-specific quality of life questionnaire will be used to measure effect of interventions [19].
- *Six-minute walk test*. The test measures the maximum distance walked after 6 min on level walkway (hallway of at least 30 m) at a quick self-paced speed, regardless of whether or not participant stops to rest [20].
- *Treadmill test (optional)*. Maximum walking distance may optionally be assessed with treadmill test at 10% slope and constantly increasing speed of up to 2 mph (3.2 km/h) over a period of 4 min and maintained maximum speed for up to 16 min. During treadmill test, participants will be blinded to the distance covered.
- *Health-related quality of life*. Health state will be derived using EQ-5D-3L descriptive system and EQ visual analogue scale [21].

Timeline of outcome assessment is specified in Table 1.

### Plans to promote participant retention and complete follow-up {18b}

Participants will only be included if they are aware of the long-time follow-up and agree to comply with the follow-up visit schedule. Upon discharge, they receive the visit schedule. At each follow-up, the site will try to contact the participant by telephone (up to three times) and, if necessary, by letter (one time) before participant is classified as lost to follow-up. If participants are unable or unwilling to on-site visits, they will be asked to answer questions by phone. If participants decline further phone contact, they will be asked to authorize release of medical information concerning safety events by their general practitioner or family members. In case all attempts fail, the site may ask the participant if he/she is willing to accept a phone call at the end of the study.

### Data management {19}

Medical data will be entered by means of an online data collection system and transmitted directly to the central data management (centre for clinical studies [ZKS] Jena). Transfer of patient-related medical data will be carried out pseudonymized. No features will be transferred that enable immediate identification of specific participants by the data management. Data entry, processing, and evaluation will comply with the provisions of the German Federal Data Protection Act (BDSG) and the EU General Data Protection Regulation (EU-GPDR). Records and documents related to the clinical trial must be kept for at least 10 years.

### Confidentiality {27}

Investigators and study staff will keep all information on participants in strict confidence. Data will be protected against unauthorized access. Appropriate local data legislation will be applied in full.

### Plans for collection, laboratory evaluation, and storage of biological specimens for genetic or molecular analysis in this trial/future use {33}

Not applicable.

## Statistical methods

### Statistical methods for primary and secondary outcomes {20a}

The primary efficacy and the primary safety endpoint will be compared by one-sided Farrington-Manning test. Noninferiority margin is set at 10% for both tests. Significance level will be set at 2.5%. Relative risk and Kaplan-Meier estimates after 1 year will be reported with 95% confidence interval (CI) for each endpoint.

Secondary continuous endpoints (ABI, walking distance test, VascuQoL, EQ-5D-3L index) will be compared by using two-sided independent samples *t*-test or non-parametric Mann-Whitney *U*-test according to the data distribution. Furthermore, the mean and standard deviation for normally distributed data or median and interquartile range otherwise will be reported for each group. Time-to event endpoints (TLR) are compared by log-rank test and Kaplan-Meier estimates with 95% CI are reported for each group after the pre-specified time points. Significance level is 5% for each test.

All analyses will be conducted in the ITT population. The primary endpoints will also be analysed in the modified ITT population, which includes only those randomized patients who received the assigned treatment.

### Interim analyses {21b}

Not applicable.

### Methods for additional analyses (e.g., subgroup analyses) {20b}

A subgroup analysis will be performed for lesion length. Cut-off to discriminate subgroups will be defined by the core laboratory.

### Methods in analysis to handle protocol non-adherence and any statistical methods to handle missing data {20c}

Patients who for any reason fail to continue in the trial until the last visit are considered dropouts. Missing values of the primary endpoints are multiply imputed to confirm the robustness of the main results.

### Plans to give access to the full protocol, participant level data, and statistical code {31c}

Not applicable.

## Oversight and monitoring

### Composition of the coordinating centre and trial steering committee {5d}

The coordinating centre consists of the principal coordinating investigator (UT, sponsor representative, Friedrich-Schiller-University Jena, Germany), the co-principal coordinating investigator (DS), the trial manager (SP), the statistician (TL), the data management team (centre for clinical studies, Jena University Hospital, Germany), and the administrative team (Department of Radiology, Jena University Hospital, Germany). Principal coordinating investigator and co-principal coordinating investigator together with the independent data and safety monitoring board (DSMB) assume the role of the trial steering committee.

### Composition of the data monitoring committee, its role, and reporting structure {21a}

The DSMB is established in order to monitor safety of participants. The DSMB consists of two independent physicians and a statistician with pertinent experience who may review study information during the conduct of the trial. Major responsibility is to make recommendations on further study conduct. Any premature termination or suspension of the trial must be discussed with the DSMB. The DSMB will review a safety event dossier, provided by the sponsor for all reported cases of severe adverse events and death. In addition, a clinical events committee (CEC) of three medical experts is established to provide an independent review of data on clinical events based on protocol-specific definitions

### Adverse event reporting and harms {22}

Adverse events are any untoward medical occurrences, unintended diseases or injuries, or any untoward clinical signs including abnormal laboratory findings in participants, users, or other persons in the context of this study, whether or not related to the investigational or control device and procedure. For users and other persons, adverse events are restricted to related adverse events. All adverse events must be specified in the study adverse event case report form. Severity and putative relationship to study devices or procedures should be noted. Investigational sites are responsible for adverse event reporting to the sponsor. Device complaints have to be reported directly to the manufacturer. Adverse device effects will be reported to the sponsor (centre for clinical studies [ZKS] Jena) quarterly.

Serious adverse events are any untoward events that occur during this study, which lead or possibly might lead, directly or indirectly, to death or serious deterioration in the state of health, life-threatening illness, injury, or permanent impairment of a body structure or a body function including chronic diseases, prolonged hospitalization, medical or surgical interventions to prevent life-threatening illness or injury, or permanent impairment to a body structure or a body function of a participant, user, or other person whether or not related to the investigational or control device and procedure. For users and other persons, serious adverse events are restricted to related serious adverse events. In the event of severe adverse events, investigational sites must immediately deliver a report to the sponsor (centre for clinical studies [ZKS] Jena, via fax within 24 h of knowledge). Any required follow-up information must be provided as soon as possible. All severe adverse events that are still ongoing at 60 months have to be followed up until resolved or until investigator confirms that no further improvement or deterioration is expected.

Incidents of any medical device with CE sign that have occurred in Germany or Austria, irrespective of a clinical study, have to be reported to the respective competent authority by the device user. The sponsor will send a quarterly report with the cumulative severe adverse event assessment to the Federal Institute for Drugs and Medical Devices (BfArM), the Austrian Federal Office for Safety in Health Care (BASG), and ethical committees involved as required.

The trial may be terminated prematurely if the DSMB or CEC raise concerns about the safety of sirolimus-coated balloon angioplasty which outweigh the current safety concerns regarding paclitaxel-coated balloon catheters. If new evidence concerning safety of paclitaxel-coated control devices will be obtained through other trials while the study is in progress, the study is being continued with the same design but with POBA as comparator.

### Frequency and plans for auditing trial conduct {23}

Inspections of the ongoing or already completed study can be carried out by the respective competent authorities in accordance with the applicable legislation. Audits serve as systematic and independent review of activities and documents related to the study to determine whether they are in accordance with the study protocol, good clinical practice (GCP), and applicable legal provisions. In addition, sponsor’s representatives can conduct monitoring and audits at participating institutions at any time as part of quality assurance.

Monitoring includes selection-, initiation-, regular on-site-, and close-out visits. Monitoring will be carried out by appropriately trained clinical research associates according to the standard operating instructions of the responsible clinical research organization (VascuScience, Leipzig, Germany). Frequency of regular and interim visits will depend on the study monitoring plan, recruitment rate, study compliance, and findings from previous visits. Principal investigators or the institutions involved will give the monitor/auditor access to all documents necessary for review.

### Plans for communicating important protocol amendments to relevant parties (e.g., trial participants, ethical committees) {25}

All substantial changes in the study protocol or other documents required for approval will be advertised to the respective competent authorities and the responsible ethics committee according to the current valid legislation at the respective time point. Implementation of a substantial amendment can only occur after formal approval of the responsible ethics committee and regulatory authority.

## Dissemination plans {31a}

Progress reports and a final report at study termination will be prepared under the responsibility of the sponsor and provided to the reviewing ethics committees as required by local regulations. Publication policy of this study has been negotiated and specified in contractual obligations and agreements between involved centres.

## Discussion

This randomized controlled study is initiated to compare efficacy and safety of femoropopliteal angioplasty by means of a novel sirolimus-coated balloon with established paclitaxel-coated balloons. All study devices are commercially available and will be used within their intended use according to manufacturer’s instruction. We hypothesize that sirolimus-coated balloon angioplasty is noninferior to paclitaxel-coated balloon angioplasty regarding both efficacy and safety.

In the last decade, endovascular revascularization has become the standard treatment for peripheral artery disease and paclitaxel-coated balloon angioplasty had been shown to be superior to POBA regarding prevention of restenosis in the femoropopliteal segment [22–27]. Therefore, the 2017 ESC guideline recommends that DCB angioplasty may be applied as “first choice” revascularization of femoropopliteal lesions < 25 cm [18]. Until recently, paclitaxel was the only drug used for peripheral DCB angioplasty. This was due to easy processing of paclitaxel for balloon coatings. High lipophilic properties assure sufficient bioavailability. Paclitaxel decreases neointimal hyperplasia by means of impairment of cellular mitosis. However, recent data from a meta-analysis [11], confirmed by findings of the nonprofit Vascular InterVentional Advances Physicians organization [28] and the US Food and Drug Administration (FDA) [29], gave rise to concerns over long-term risk of mortality due to possible side effects of paclitaxel. However, a causal relationship between cytotoxic properties of paclitaxel and increased long-term mortality after treatment with paclitaxel-coated devices had not been demonstrated. On the other hand, recent large-scale studies based on patient-level data found no evidence for increased long-term mortality related to paclitaxel [12, 13]. Moreover, an unplanned interim analysis of a large Swedish randomized registry-based study (SWEDEPAD trial) revealed no difference between paclitaxel-coated and uncoated devices concerning all-cause mortality after endovascular interventions through an average follow-up of 2.5 years [30].

Against the background of conflicting evidence on safety of paclitaxel, the cytostatic drug sirolimus could be an alternative. Recently, coating technologies were adapted to the less lipophilic sirolimus by complex engineering. The active ingredient sirolimus is intended to prevent restenosis through its immunosuppressive and antiproliferative properties. In the polymer-free Magic Touch® PTA sirolimus-coated balloon catheter, sub-micron sized sirolimus particles are encapsulated into highly biocompatible phospholipid carriers (excipient) which improve both bioavailability of sirolimus and adhesion to the balloon surface and thus, prevention of drug loss (“wash-off”) into the blood stream and possible adverse downstream effects. Upon balloon inflation drug carrier and sirolimus are transferred into the arterial wall. Subsequently, upon pH-change, the phospholipid excipient releases sirolimus which penetrates into deeper vessel layers where it is retained long enough for sufficient neointimal inhibition. Effective drug transfer allows a relative low drug dose density of sirolimus-coated balloons. Additionally, the comparably low tissue retention and the broad therapeutic range of sirolimus decrease the risk of local vessel wall toxicity [31].

A pilot pre-market study on sirolimus-coated balloon angioplasty (XTOSI pilot study on MagicTouch® PTA sirolimus-coated balloon) revealed promising 6-month results of 88.2% primary patency for femoropopliteal and 74.0% for infrapopliteal lesions. Freedom from major adverse events was 89.5% for participants with femoropopliteal and 84.0% for those with infrapopliteal lesions (Chok E, AMP symposium 2020). In addition, first-in-human outcomes from a small-scale study were presented from the polymer-based SELUTION sustained-limus-release™ drug-coated balloon for the treatment of intermittent claudication. The primary outcome of late lumen loss at six months was significantly lower than the optimal performance outcome value of POBA. Six-month primary patency was 88.4% [32, 33].

Effect size of DCB angioplasty depends not only on the type of coating but also on the treatment strategy such as pre-dilation and bailout stenting, as well as on lesion complexity [16]. Thus, we opted for a randomized study design with well-balanced lesion- and procedure-related preconditions to ensure a fair head-to-head comparison of sirolimus- and paclitaxel-coated balloon angioplasty.

If our study demonstrates noninferiority of the MagicTouch® PTA sirolimus-coated balloon to paclitaxel-coated balloons regarding efficacy and safety, the device can serve as a viable non-paclitaxel option for the treatment of femoropopliteal lesions.

## Abbreviations

ABI: Ankle brachial index
CEC: Clinical event committee
DCB: Drug-coated balloon
DSMB: Data safety and monitoring board
CLTI: Chronic limb-threatening ischemia
DAPT: Dual antiplatelet therapy
DUS: Duplex ultrasound
EQ-5D-3L: European Quality of Life 5 Dimensions 3 Level Instrument
PAD: Peripheral artery disease
POBA: Plain old balloon angioplasty
PSVR: Peak systolic velocity ratio
PTA: Percutaneous transluminal angioplasty
VascuQoL: Vascular quality of life questionnaire
